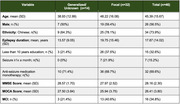# Suboptimal seizure control is associated with increased risk of MCI among Adult Patients with Epilepsy

**DOI:** 10.1002/alz.088935

**Published:** 2025-01-03

**Authors:** Jia Yi Shen, Chirin Soh, Wee Jin Chew, Rachel Siew, Seyed Ehsan Saffari, Pei Xuan Koh, Yee Leng Tan, Sheila D/O Srinivasan, Nigel Choon Kiat Tan, Ngai Kun Loh, Louis CS Tan, Kok Pin Ng

**Affiliations:** ^1^ Department of Neurology, National Neuroscience Institute, Singapore, Singapore, Singapore Singapore; ^2^ Yong Loo Lin School of Medicine, National University of Singapore, Singapore, Singapore Singapore; ^3^ Health Services and Systems Research, Duke‐NUS Medical School, Singapore, Singapore Singapore

## Abstract

**Background:**

Epilepsy is associated with increased risk for dementia, which adversely impacts the quality of life for patients and their families. Mild cognitive impairment (MCI) is the prodromal stage of dementia offering an important window for intervention. However, the epilepsy related risk factors for MCI are not well understood. The ongoing Mild Cognitive Impairment among Adult Patients with Epilepsy (MCAPE) study is a longitudinal study evaluating the prevalence and risk factors for MCI amongst adult patients with epilepsy (PWE). This abstract summarizes the interim findings.

**Method:**

Adult PWE were recruited from outpatient clinics at a tertiary centre, the National Neuroscience Institute, Singapore. Patients with previous stroke, known dementia, or intellectual disability were excluded. Participants underwent a self‐administered questionnaire on their lifestyle risk factors and epilepsy‐specific factors, followed by cognitive testing with the Mini Mental State Assessment (MMSE) and Montreal Cognitive Assessment (MOCA). A MOCA cut‐off of <27 was used to diagnose MCI. Logistic regressions evaluated the associations of clinical risk factors with presence of MCI, corrected for age and education as confounders.

**Result:**

Forty‐six participants were recruited and completed cognitive testing. Twenty‐three participants (50.0%) had subjective cognitive complaints. Cognitive assessment revealed that 16 participants (34.8%) had MCI. Among epilepsy factors, multivariate logistic regression found that a seizure frequency of once a month or more was strongly associated with increased risk of MCI (OR 109, p = 0.004). Epilepsy subtype, duration and use of multiple anti‐seizure medications were not significantly associated with MCI.

**Conclusion:**

This pilot cross‐sectional study showed that MCI is a significant problem among PWE, and that suboptimal seizure control was associated with increased risk of MCI. Prospective studies are need to determine effective interventions to mitigate MCI among PWE.